# Supplementary material for: Integrated Spectroscopic Analysis of Wild Beers: Molecular Composition and Antioxidant Properties
Source: Int J Mol Sci. 2025 Jul 21;26(14):6993. doi: 10.3390/ijms26146993 (PMC12294954; doi:10.3390/ijms26146993)
Supplement: Supplementary file 1 [file ijms-26-06993-s001.zip › ijms-3760064-supplementary.pdf]

Integrated Spectroscopic Analysis of Wild Beers: Molecular Composition and Antioxidant Properties

Dessislava Gerginova [1,2], Plamena Staleva [1,3], Zhanina Petkova [1,2], Konstantina Priboyska [1], Plamen Chorbadzhiev [1,4], Ralitsa Chimshirova [1,2], Svetlana Simova [1,2,]*

[1] Institute of Organic Chemistry with Centre of Phytochemistry, Bulgarian Academy of Sciences, Acad. G. Bonchev str. Bl. 9, 1113 Sofia, Bulgaria; dessislava.gerginova@orgchm.bas.bg (D.G.), plamena.staleva@orgchm.bas.bg (P.S.), zhanina.petkova@orgchm.bas.bg (Z.P.), konstantina.priboyska@orgchm.bas.bg (K.P.), plamen.chorbadzhiev@orgchm.bas.bg (P.C.), ralitsa.chimshirova@orgchm.bas.bg (R.C.).

[2] Centre of Competence "Sustainable Utilization of Bio-Resources and Waste of Medicinal and Aromatic Plants for Innovative Bioactive Products" (BIORESOURCES BG), 1000 Sofia, Bulgaria.

[3] Laboratory for Extraction of Natural Products and Synthesis of Bioactive Compounds, Research and Development and Innovation Consortium, Sofia Tech Park JSC, 111 Tsarigradsko Shose Blvd., 1784 Sofia, Bulgaria.

[4] Faculty of Chemical and Systems Engineering, University of Chemical Technology and Metallurgy, 8 St Kliment Okhridski blvd, 1756 Sofia, Bulgaria.

* Correspondence: svetlana.simova@orgchm.bas.bg (S.S)

SUPPORTING INFORMATION

LIST OF TABLES:

Table S1. List of wild ale samples analyzed, including beer (name & brewery), added ingredients, aging, country of origin, and alcohol by volume (ABV, %).
Table S2. Presence (+) and absence (-) in the wild ales of the 62 compounds, identified by LC-MS.
Table S3. Misclassification tables from OPLS-DA models used to discriminate (a) added fruits, (b) aging duration, and (c) country of origin

LIST OF FIGURES:

Figure S1. Linear regression plots comparing quantification by NMR and relative intensity data from LC-MS analysis: (a) malic acid, (b) citric acid, (c) gallic acid, and (d) tartaric acid.
Figure S2. Receiver operating characteristic (ROC) curves and AUC for OPLS-DA models used to classify wild ales by (a) added fruits, (b) aging duration, and (c) country of origin.
Figure S3. Loading plots from the OPLS-DA models used to classify wild ales according to (a) added fruits, (b) aging duration, and (c) country of origin.
Figure S4. Variable Importance in Projection (VIP) scores from the OPLS-DA models for (a) added fruits, (b) aging duration, and (c) country of origin. Error bars represent cross-validation variability.

Table S1. List of wild ale samples analyzed, including beer (name & brewery), added ingredients, aging, country of origin, and alcohol by volume (ABV, %).

	Beer (Name & *Brewery*)	Added ingredients	Aging	Country of origin	ABV%
**W1**	Crest –*Pinta Barrel Brewing*	Strawberries	12 months	Poland	5.5
**W2**	Impulse –*Pinta Barrel Brewing*	Gooseberries	12 months	Poland	6
**W3**	After Hours: Red Wild Ale –*Pinta Barrel Brewing*	Red grapes, raspberry, shadbush	12 months	Poland	6.5
**W4**	After Hours: Citrus Wild Ale–*Pinta Barrel Brewing*	Mandarin, orange, lime	12 months	Poland	6
**W5**	Arana Blues –*Attik Brewing/Cervesa Guineu*	Blueberries, plums	24 months (American oak)	Spain	5.5
**W6**	Salvatik–*Attik Brewing/Cervesa Guineu*	None listed	24 months (American oak)	Spain	5
**W7**	Blossom –*Attik Brewing/Cervesa Guineu*	Picota cherries	24 months (American oak)	Spain	5.2
**W8**	Bacchanalia Pinot Noir – *Beer Bastards*	Pinot Noir	36 months french Oak	Bulgaria	6
**W9**	Bacchanalia Riesling – *Beer Bastards*	Riesling	36 months french Oak	Bulgaria	6
**W10**	Timeless –*White Stork Beer Co*	Bulgarian spelt, rye	25 months (French oak)	Bulgaria	5.9
**W11**	Meander (Batch No. 5) – *Nevel*	Rhubarb, blackcurrant leaf	18 months	Netherlands	6.3
**W12**	Bast (Batch No. 4) – *Nevel*	Spruce, blackthorn, pine, birch bark	14 months	Netherlands	5.2
**W13**	Mijmer (Batch No. 3) – *Nevel*	Blackcurrant, fennel seed	24 months	Netherlands	5
**W14**	Halm (Batch No. 1) – *Nevel*	Mugwort	7 months	Netherlands	5.3
**W15**	Purper (Batch No. 4) – *Nevel*	Buckwheat, lavender, aronia	4 months	Netherlands	4.5
**W16**	Dauw (Batch No. 4) – *Nevel*	Elderflower, green pepper, citrus	26 months	Netherlands	5.4
**W17**	Gloed (Batch No. 7) – *Nevel*	Cherries, blackcurrants	14 months	Netherlands	6.2
**W18**	Trial & Terroir: Raspberry 2019 –*Hop Hooligans*	Raspberries	36 months (bourbon & sherry barrels)	Romania	6
**W19**	Trial & Terroir: Fragola Grape 2019 –*Hop Hooligans*	Fragola grape must	36 months (bourbon & sherry barrels)	Romania	7

**W20**	Symbol of Renaissance 2021–*Baghaven Brewing and Blending*	Yellow peaches	18-36 months	Denmark	8.2
**W21**	Reflections of Matter 2020 –*Baghaven Brewing and Blending*	Raspberries, apricots, Cabernet Sauvignon	18–36 months	Denmark	6
**W22**	Rubus of Rose Vanilje 2021–*Baghaven Brewing and Blending*	Raspberries, vanilla bean	24–36 months	Denmark	6.1

Table S2. Presence (+) and absence (-) in the wild ales of the 62 compounds, identified by LC-MS *

Compound	W1	W2	W3	W4	W5	W6	W7	W8	W9	W10	W11	W12	W13	W14	W15	W16	W17	W18	W19	W20	W21	W22
**1**	+	+	+	+	+	+	+	+	+	+	+	+	+	+	+	+	+	+	+	+	+	+
**2**	+	+	+	+	+	+	+	+	+	+	+	+	+	+	+	+	+	+	+	+	+	+
**3**	+	+	+	+	+	+	+	+	+	+	+	+	+	+	+	+	+	+	+	+	+	+
**4**	+	+	+	+	+	+	+	+	+	+	+	+	+	+	+	+	+	+	+	+	+	+
**5**	+	+	+	+	+	+	+	+	+	+	+	+	+	+	+	+	+	+	+	+	+	+
**6**	+	+	+	+	+	+	+	+	+	+	+	+	+	+	+	+	+	+	+	+	+	+
**7**	+	+	+	+	+	+	+	+	+	+	+	+	+	+	+	+	+	+	+	+	+	+
**8**	-	+	+	+	+	+	+	+	+	+	+	+	+	+	+	+	+	+	+	+	+	+
**9**	+	+	+	+	+	+	+	-	-	+	+	-	+	+	+	+	+	+	-	+	+	+
**10**	+	+	+	+	+	+	+	+	+	+	+	+	+	+	+	+	+	+	+	+	+	+
**11**	+	-	+	+	+	-	-	-	-	+	-	+	-	-	-	-	-	-	-	-	+	-
**12**	+	+	+	-	-	-	-	+	+	+	-	+	-	-	+	-	+	-	+	+	+	+
**13**	+	+	+	+	+	+	+	+	+	+	+	+	+	+	+	+	+	+	+	+	+	+
**14**	+	+	+	+	+	-	+	-	-	-	+	-	+	-	+	-	+	-	-	+	+	-
**15**	-	+	+	+	+	-	-	-	-	-	+	-	+	-	+	-	+	-	-	-	-	+
**16**	+	+	-	+	+	+	+	+	+	+	+	+	+	+	+	+	+	+	+	+	+	+
**17**	+	+	+	+	-	-	-	-	-	-	-	-	+	-	+	+	-	-	-	+	+	+
**18**	+	+	+	+	+	+	+	+	+	+	+	+	+	+	+	+	+	+	-	+	+	+
**19**	+	+	+	+	+	+	+	-	-	+	+	+	+	+	+	+	+	-	-	+	+	+
**20**	+	+	+	+	+	+	+	+	+	+	+	+	+	+	+	+	+	+	+	+	+	+
**21**	+	+	+	+	+	+	+	+	+	+	+	+	+	+	+	+	+	+	+	+	+	+
**22**	+	+	+	-	+	-	+	-	-	-	+	-	+	-	+	-	+	-	-	-	+	+
**23**	+	-	+	+	+	-	+	+	-	+	+	-	+	+	+	+	+	+	+	+	+	-
**24**	+	+	-	-	+	+	+	+	+	+	+	+	+	-	+	-	+	+	+	+	+	+
**25**	-	-	+	-	+	-	+	-	-	-	+	-	-	-	+	+	-	-	-	+	-	-
**26**	+	-	+	+	-	-	-	-	-	+	+	+	-	+	+	+	-	-	-	-	-	-

**27**	+	-	+	+	-	-	+	-	-	+	+	+	-	+	+	+	-	-	+	-	-	-
**28**	-	-	+	-	-	-	-	-	-	+	-	-	-	-	+	-	+	-	-	-	-	+
**29**	+	+	+	+	+	+	+	+	+	+	+	+	+	+	+	+	+	+	+	+	+	+
**30**	+	-	+	-	+	-	-	-	-	+	+	+	-	+	-	-	-	-	-	-	-	-
**31**	+	+	+	-	-	-	-	-	-	-	+	-	+	-	+	+	+	+	+	-	-	-
**32**	-	-	-	-	-	-	-	-	-	-	-	-	+	-	+	-	+	-	-	-	-	-
**33**	+	-	-	-	+	+	+	-	-	+	+	-	-	+	-	+	-	-	-	-	-	-
**34**	+	+	+	-	+	+	+	+	+	+	+	+	+	+	+	+	+	+	+	+	+	+
**35**	-	-	+	-	-	-	-	-	-	-	-	-	-	-	-	-	-	-	-	-	-	-
**36**	-	-	+	+	+	+	+	-	-	+	+	+	-	+	+	-	+	-	-	-	+	+
**37**	+	-	+	+	+	+	+	-	-	+	+	+	-	+	+	+	+	-	-	-	-	+
**38**	+	-	+	+	+	+	+	-	-	+	-	+	-	+	-	+	-	-	-	-	-	-
**39**	-	-	+	-	+	-	-	-	-	-	+	+	-	-	+	-	-	-	-	-	+	+
**40**	-	-	+	-	-	-	+	-	-	-	+	-	+	+	+	+	-	-	-	-	-	-
**41**	+	-	+	+	+	+	+	-	-	+	+	+	-	+	-	+	-	-	-	-	-	-
**42**	-	-	+	-	-	-	+	-	-	-	-	-	-	+	-	+	+	-	-	-	-	-
**43**	-	-	-	+	-	-	-	-	-	-	-	-	-	-	-	-	-	-	-	-	-	-
**44**	-	-	-	-	-	-	-	-	-	-	-	-	-	-	+	-	+	-	-	-	-	-
**45**	+	+	+	+	+	+	+	+	+	+	+	+	+	+	+	+	+	+	+	+	+	+
**46**	+	+	+	+	+	-	-	+	+	+	+	+	+	+	+	+	+	-	-	+	+	+
**47**	-	+	-	-	-	-	+	+	-	+	+	+	+	+	+	-	+	+	-	-	+	+
**48**	+	+	+	+	+	+	+	+	+	+	+	+	+	+	+	+	+	+	-	+	+	+
**49**	+	+	+	+	-	-	-	+	+	+	+	-	+	-	+	-	+	-	+	+	+	+
**50**	+	+	+	+	+	+	+	+	+	+	+	+	+	+	+	+	+	+	+	+	+	+
**51**	+	+	+	+	-	+	+	+	+	+	+	+	+	+	+	+	+	-	+	+	+	+
**52**	+	+	+	+	+	+	+	+	+	+	+	+	+	+	+	+	+	+	+	+	+	+
**53**	+	+	+	+	-	+	-	+	+	+	+	+	+	+	+	+	+	+	+	+	+	+
**54**	+	+	+	-	+	+	-	+	+	+	+	+	+	+	+	+	+	+	-	+	+	+

**55**	+	+	+	+	+	+	+	+	+	+	+	+	+	+	+	+	+	+	+	+	+	+
**56**	+	-	+	+	-	-	-	+	+	+	+	+	+	+	-	+	-	-	+	-	-	-
**57**	-	-	+	+	-	-	-	+	-	+	-	+	-	-	-	+	-	-	-	-	-	-
**58**	+	+	+	+	+	+	+	+	+	-	+	+	+	+	+	+	+	+	+	+	+	+
**59**	+	+	+	+	+	+	+	+	+	+	+	+	+	+	+	+	+	+	+	+	+	+
**60**	+	+	+	+	+	+	+	+	+	+	+	+	+	+	+	+	+	+	+	+	+	+
**61**	-	+	+	+	-	-	-	+	+	+	-	+	+	+	+	+	+	-	-	-	+	-
**62**	-	-	+	+	-	+	-	+	+	-	-	+	-	-	-	-	-	-	-	-	-	-

* Chemical formula of compounds **1-62** are provided in Table 2 of the main manuscript.

Table S3. Misclassification tables from OPLS-DA models used to discriminate (a) added fruits, (b) aging duration, and (c) country of origin

(a)	Members	Correct	Dark Fruits	Light Fruits	No Fruits
**Dark Fruits**	13	100%	13	0	0
**Light Fruits**	4	100%	0	4	0
**No Fruits**	5	100%	0	0	5
**Total**	22	100%	13	4	5

(b)	Members	Correct	Up to 18 m	More than 18 m
**Up to 18 m**	9	100%	9	0
**More than 18 m**	13	100%	0	13
**Total**	22	100%	9	13

(c)	Members	Correct	PL	ES	BG	NL	RO	DK
**PL**	4	100%	4	0	0	0	0	0
**ES**	3	100%	0	3	0	0	0	0
**BG**	3	100%	0	0	3	0	0	0
**NL**	7	100%	0	0	0	7	0	0
**RO**	2	100%	0	0	0	0	2	0
**DK**	3	100%	0	0	0	0	0	3
**Total**	22	100%	4	3	3	7	2	3

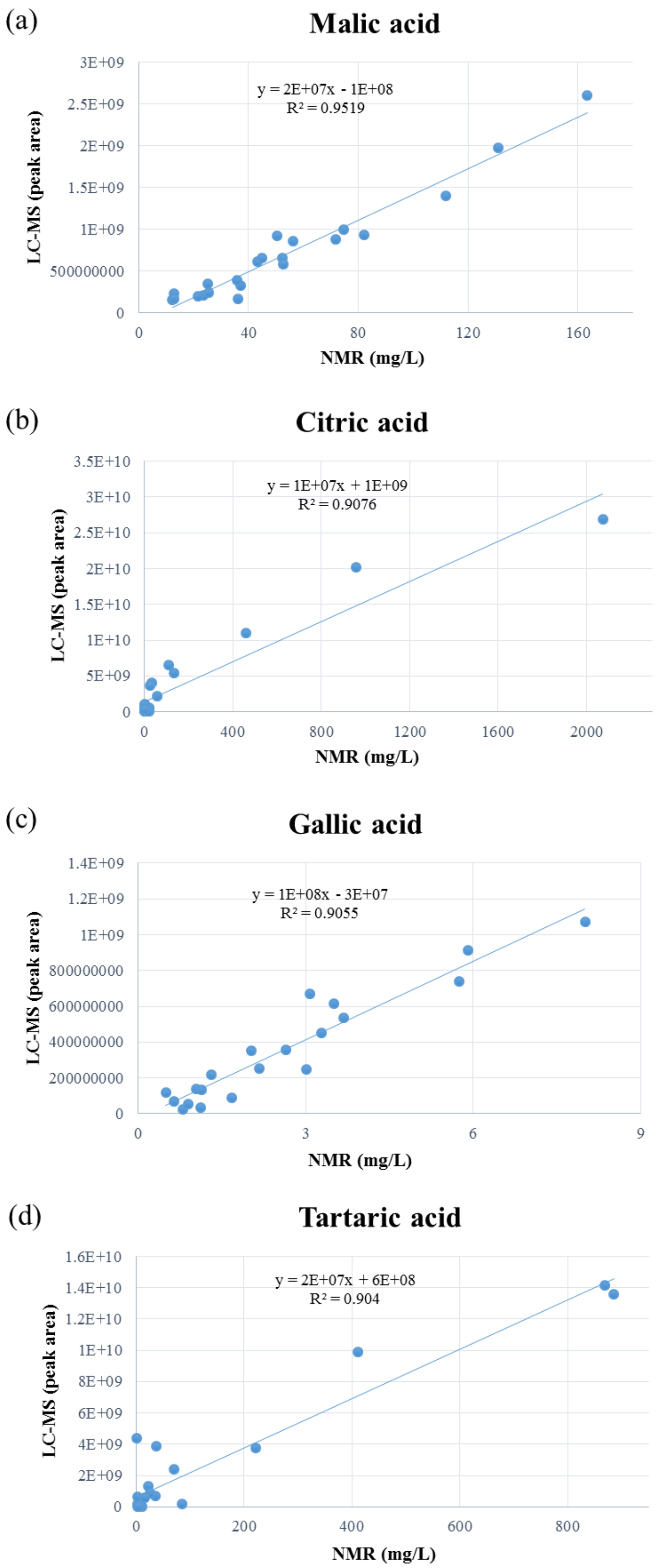

Figure S1. Linear regression plots comparing quantification by NMR and relative intensity data from LC-MS analysis: (a) malic acid, (b) citric acid, (c) gallic acid, and (d) tartaric acid

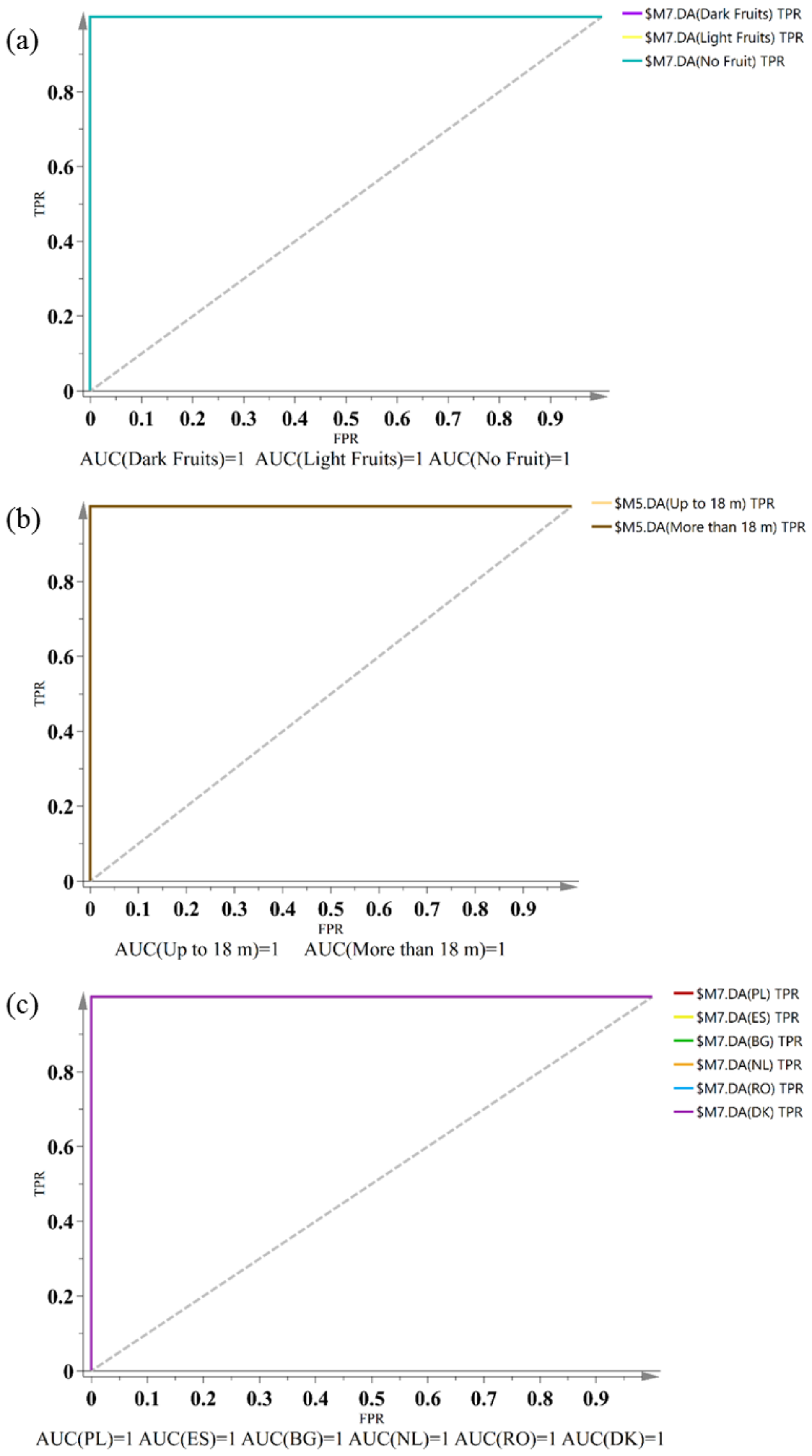

Figure S2. Receiver operating characteristic (ROC) curves and AUC for OPLS-DA models used to classify wild ales by (a) added fruits, (b) aging duration, and (c) country of origin

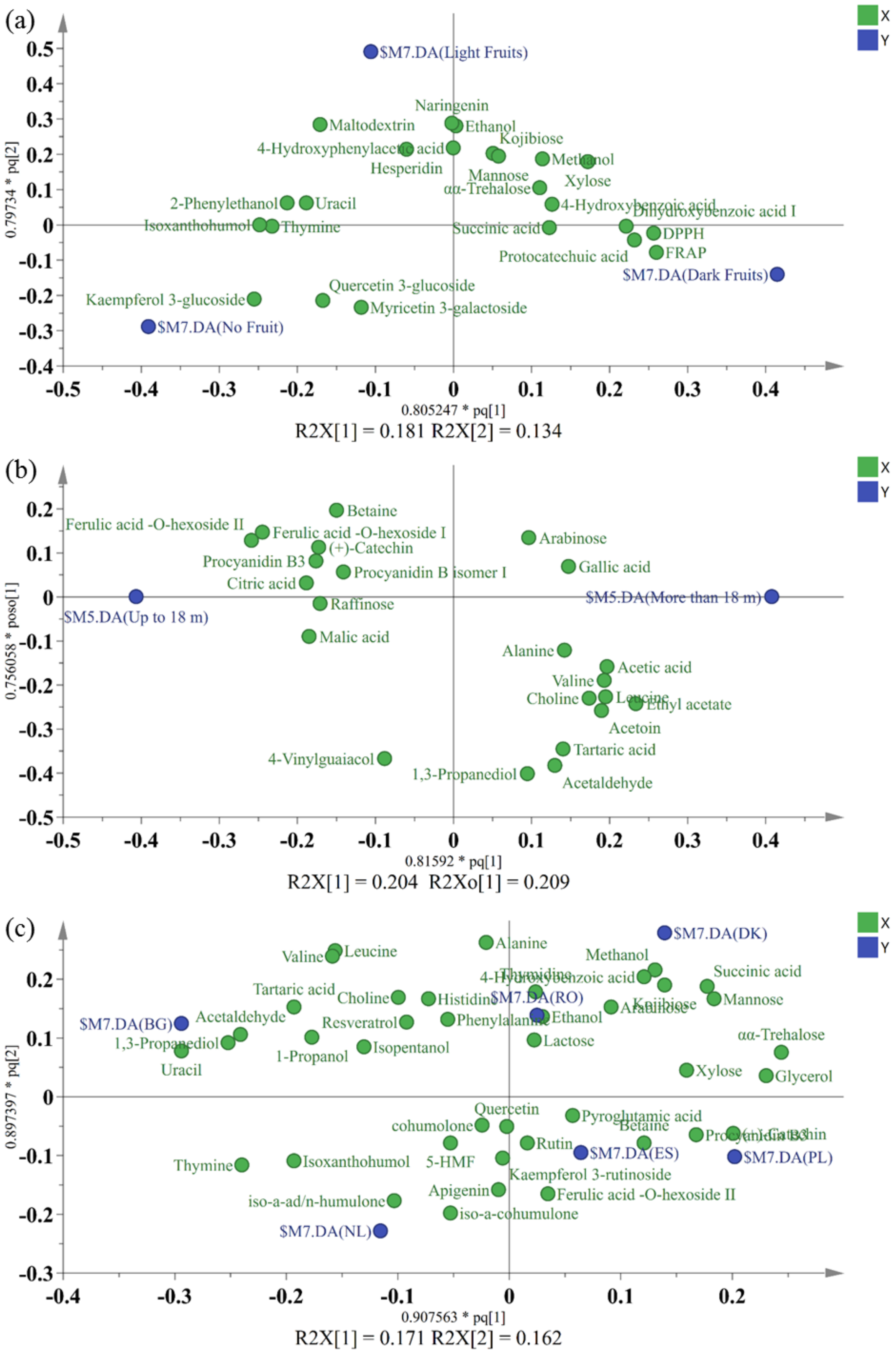

Figure S3. Loading plots from OPLS-DA models used to classify wild ales according to (a) added fruits, (b) aging duration, and (c) country of origin.

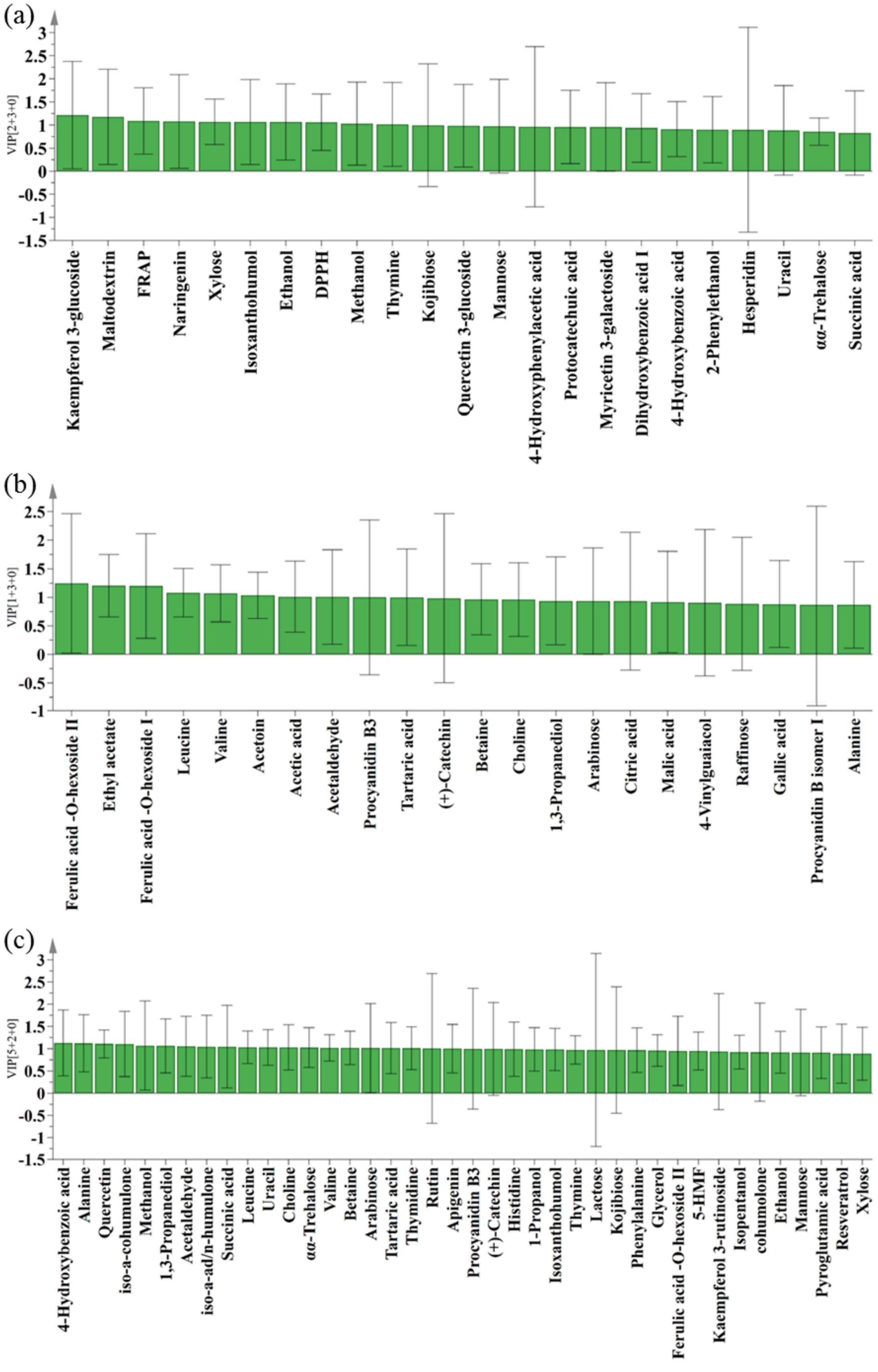

Figure S4. Variable Importance in Projection (VIP) scores from the OPLS-DA models for (a) added fruits, (b) aging duration, and (c) country of origin. Error bars represent cross-validation variability.
